# Tunable Heat-Flux Rectification in Graded Nanowires in Non-Linear Guyer-Krumhansl Regime

**DOI:** 10.3390/nano13091442

**Published:** 2023-04-23

**Authors:** Isabella Carlomagno, Vito Antonio Cimmelli, David Jou

**Affiliations:** 1 Department of Industrial Engineering, University of Salerno, Via Giovanni Paolo II, 132, 84084 Fisciano, Italy; 2 Department of Mathematics, Computer Science and Economics, University of Basilicata, Viale dell’Ateneo Lucano, 10, 85100 Potenza, Italy; 3 Departament de Física, Universitat Autònoma de Barcelona, 08193 Bellaterra, Catalonia, Spain; 4 Institut d’Estudis Catalans, Carme 47, 08001 Barcelona, Catalonia, Spain

**Keywords:** heat rectification, graded materials, composition-dependent thermal conductivity, nonlinear Guyer-Krumhansl equation

## Abstract

We study heat rectification in composition-graded nanowires, with nonlocal and nonlinear effects taken into account in a generalized Guyer-Krumhansl equation. Using a thermal conductivity dependent on composition and temperature, the heat equation is solved. Introducing a non-vanishing heat supply (as for instance, a lateral radiative heat supply), we explore the conditions under which either nonlocal or nonlinear effects or both contribute to heat rectification and how they may be controlled by means of the external radiative flux. The corresponding rectification coefficients are calculated as well, and the physical conditions under which the system becomes a thermal diode are pointed out.

## 1. Introduction

Heat transport theory is currently experiencing a true revolution since new phenomenologies, due to miniaturization, have been discovered [1,2]. The new phenomena depend on the relationship between the mean free path of the heat carriers *ℓ*, and the characteristic dimension of the conductor L, expressed by the Knudsen number Kn=ℓ/L. In classical heat conduction theory ℓ/L≪1. However, Kn can be incremented by a reduction of L, as in miniaturization technology. In the last few decades, nanosystems have been widely applied in such a technology. The word nanosystem means a systems with at least one dimension at the nanoscale. They provide an interesting avenue to obtain highly performing devices, for example, by making nanocomposites, adding nanoparticles to a bulk material, or using one-dimensional nanostructures. Currently, the research on nanotechnology involves the preparation of different types of nanomaterials and the analysis of their properties for applications in medical technology, microelectronics, aerospace, energy production and management, and biotechnology [3,4,5,6,7]. The main reason of such a wide field of application is that recent industrial techniques allow to modify the properties of nanomaterials in order to adapt them for several applications.

The system considered here is a rigid nanowire of length L, with composition varying along its length. More precisely, we consider a silicon-germanium alloy, which will be denoted by ${\mathrm{Si}}_{c}{\mathrm{Ge}}_{1-c}$, with the stoichiometric variable *c* dependent of the position *x* of the points of the system, and such that c(0)=0 and c(L)=1. Thus, for x=0 we have pure germanium, and for x=L we have pure silicon. In the intermediate points, namely for x∈[0,L], the composition *c* depends on the function c(x), which is determined while manufacturing the material. The easiest situation, which will be considered in the present paper, is the linear dependency on *x*, namely c=x/L. Different graduation laws have been analyzed in Ref. [8]. Among the many practical applications of ${\mathrm{Si}}_{c}{\mathrm{Ge}}_{1-c}$ alloys, of particular interest in micro/nano-electronics is the heat rectification, namely, the fact that the same temperature gradient but acting in opposite directions on a given system yields different values of the corresponding heat flux [9].

**Figure 1:**
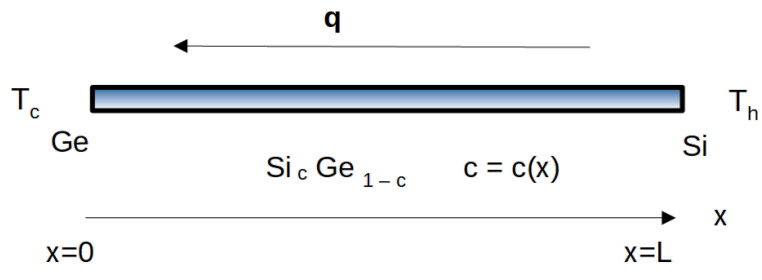
Sketch of a ${\mathrm{Si}}_{c}{\mathrm{Ge}}_{1-c}$ nanowire, of length L, with the difference in temperature $T_{h}-T_{c}$ at its ends.

In Figure 1 it is sketched a ${\mathrm{Si}}_{c}{\mathrm{Ge}}_{1-c}$ nanowire, of length L, with the difference in temperatures $T_{h}-T_{c}$ ($T_{h}$ hottest temperature, $T_{c}$ coldest temperature) at its ends.

Thermal rectification is an asymmetric process in which the thermal properties of the material along a specific axis depend on the direction of the temperature gradient or the heat current. Thus, modern designs for thermal rectification are based on the different temperature dependences of the thermal conductivities of different bulk materials that are used. Meanwhile, there are several experimental and theoretical studies attempting to understand the thermal rectification mechanisms. In fact, an improved understanding of how thermal rectification is achieved is useful in the development of devices such as thermal transistors, thermal logic circuits, and thermal diodes, which are utilized in micro/nano-electronic cooling as well as in thermal memory and computations. Several mechanisms for thermal rectification, including surface roughness/flatness at material contacts, a thermal potential barrier between material contacts, and adifference in temperature dependence of thermal conductivity between dissimilar materials at a contact, have been discovered [10]. A promising field of research in heat rectification regards the design and realization of thermal diodes, namely, devices whose thermal resistance for heat flow in one direction is extremely stronger than that for heat flow in the opposite direction. In more detail, when a thermal diode’s terminal is hotter than a second terminal, heat flows easily from the first to the second, but when the second terminal is hotter than the first, a negligible quantity of heat flows from the second to the first. In practice, when the diode’s first terminal is at a higher temperature than the second terminal, heat is allowed to flow freely from the first terminal to the second terminal. In contrast, when the diode’s second terminal is at a higher temperature as compared with the first terminal, heat flow from the second terminal to the first one is strongly reduced [11].

**Figure 2:**
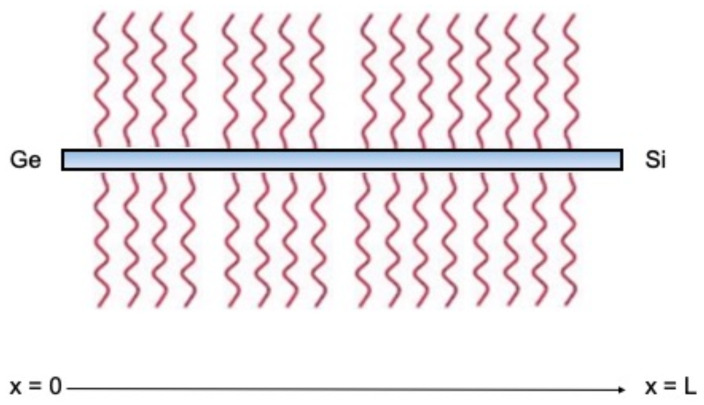
Sketch of a nanowire of length L immersed in a field of thermal radiation.

Rectification of the heat flux has been extensively studied by the authors in [8,9,12,13,14,15,16]. In those papers, the classical Fourier heat-transport equation has been used. It is worth observing that such an equation was semilinear, i.e., it was linear with respect to the temperature gradient but nonlinear with respect to the dependency of the thermal conductivity on composition and temperature. Such an equation allows the existence of regular solutions (see Ref. [15] and Figure 2 therein). In the present research, we go a step further, by applying a genuinely nonlinear (i.e., nonlinear with respect to the heat flux) Guyer-Krumhansl equation, in order to investigate the effects of the nonlinearity of the heat equation with respect to the heat flux too. Furthermore, the consequences of the presence in the heat equation of some nonlocal terms, i.e., some quantities that depend on the long-distance interactions of the heat carriers, are explored as well. In Ref. [16] we have proved that, in the case of thermoelastic systems such as silicon thin films and graphene ribbons, heat rectification can be tuned by applying an external mechanical stress. This seems to be an important property, allowing a further control of the rectification properties beyond the one due to the varying composition. Indeed, although composition is a precious tool for controlling rectification, it cannot be changed once the system has been manufactured. The external stress, instead, can be changed and adapted to the different needs.

Here we consider a different way to control the rectification of the heat flux, namely, by heating the internal points of the body by a heat source ρr, with ρ as the mass density. Coleman and Noll, in their celebrated article on the thermodynamics of elastic materials [17], call *r* “density of absorbed radiation”, and claim that r(x,t) is “the heat supply per unit mass and unit time absorbed by the material and furnished by radiation from the external world”. One of the possibilities for obtaining such an additional heat supply is to put the system in a radiation field [18]. At the nanoscale, radiative heat can be supplied to the system by placing it in an electromagnetic radiation generator. Figure 2 schematizes a nanowire of length L immersed in a field of thermal radiation.

The system under consideration is a composition-graded ${\mathrm{Si}}_{c}{\mathrm{Ge}}_{1-c}$ wire, of length L=100nm (nanowire) and L=3mm. In this way, we can investigate the differences in the physical behavior of the systems due to their different lengths (size effects). It is worth noticing that such a choice is based on the experimental data at our disposal, which have been obtained for systems of length L=100nm, and L=3mm [19,20].

Under the hypothesis that a radiative heat supply per unit of volume and time is provided to the internal points of the system, we explore heat rectification by:

(1) applying a direct heat flux $q_{d}$ on the Ge side at temperature $T_{h}$ (hottest T), and obtaining the corresponding temperature $T_{c}$ (coldest T) at the Si side (direct situation);
(2) applying different values of the reverse heat flux on the Si side at the highest temperature $T_{h}$ until finding the value $q_{r}$ (reverse heat flux) for which the temperature at the Ge side is the lowest temperature $T_{c}$ previously obtained for $q_{d}$ (reverse situation);
(3) calculating the rectification coefficient as $R\equiv q_{r}/q_{d}$.

In the direct and reverse processes, the radiative heat supply is the same along the system. If R<1, we may conclude that the difference in temperature $T_{h}-T_{c}$ applied from silicon to germanium produces a heat flux that is smaller with respect to that produced by the same difference in temperature applied from germanium to silicon. If, instead, R>1, we may conclude that the difference in temperature $T_{h}-T_{c}$ applied from silicon to germanium produces a heat flux that is greater with respect to that produced by the same difference in temperature applied from germanium to silicon. In particular, if R≃0, then the difference in temperature $T_{h}-T_{c}$ does not produce heat flow from silicon to germanium, i.e., heat can only flow from germanium to silicon. Such a property allows the design of the so-called thermal diodes, whose thermal resistance for heat flow in one direction is extremely stronger than that for heat flow in the opposite direction. The result of our exploration is that, in principle, both nonlocal and nonlinear effects may influence the rectification of the heat flux, and such an influence depends on the strength of the direct heat flux $q_{d}$. The paper runs as follows.

In Section 2, we discuss the main features of nonlocal and nonlinear heat transport at the nanoscale and illustrate the different governing equations for the heat flux.

In Section 3, we present the physical model for a one-dimensional system and point out the conditions under which one of the following situations may occur: (1) only nonlocal effects are present; (2) both nonlocal and nonlinear effects are present; (3) neither nonlocal nor nonlinear effects are present.

In Section 4, for a composition graded alloy of length L=100nm, and L=3mm, at T=300K, we calculate the rectification coefficient when some of the situations pointed out in Section 3 occur. The temperature profiles are calculated as well. The results are discussed in light of the material properties and the value of the direct heat flux.

In Section 5, concluding remarks, together with a discussion on possible developments of the present research, are pointed out.

In Appendix A, starting from the experimental data in [19,20], we obtain a mathematical representation of the thermal conductivity as a function of composition and temperature, in a neighborhood of three reference temperatures, namely, T=300K, T=400K and T=500K.

## 2. Nonlocal and Nonlinear Heat Transport at Nanoscale

The classical Fourier’s law [21]

(1) q=−λ∇T,

where q is the heat flux, λ the thermal conductivity, and T the temperature, is valid when ℓ/L≪1, namely, for ℓ≪L. When the mean free path of the heat carriers is comparable to the characteristic dimension of the conductor, i.e., Kn≃1, more complicated transport laws for the heat flux are necessary [22,23].

Nowadays, there is a current interest in phonon hydrodynamics, a mesoscopic approach to heat-conduction where the heat carriers are regarded as a fluid, whose hydrodynamic equations of motion describe the transport of heat [24].

The phonon hydrodynamics leads to the Guyer-Krumhansl transport equation for the heat flux q, i.e.,

(2) $$\tau_{R}\frac{\partial q}{\partial t}+q=-\lambda \nabla T+\ell^{2}\left(\nabla^{2}q+2\nabla \nabla \cdot q\right),$$

where $\tau_{R}$ is the relaxation time due to the resistive (quasi-momentum not conserved) scattering of phonons in the bulk. Moreover, the thermal conductivity $\lambda =\varrho c_{v}\tau_{R}{\bar{v}}^{2}/3$, where ϱ is the mass density, $c_{v}$ the specific heat per unit mass at constant volume, and $\bar{v}$ is the average of the phonons’ speed, is constant [25,26,27]. Phonon hydrodynamics comes from kinetic theory, which is able to keep up with the pace of current macroscopic searches [24], and is compatible with generalized formulations thermodynamics beyond local-equilibrium.

Phonons are quasi-particles generated by the crystal oscillations, following the Bose-Einstein statistics. Indeed, there are two modes of vibration of atoms in crystals, namely, longitudinal vibrations and transversal vibrations. In the longitudinal mode, the displacement of atoms from their positions of equilibrium coincides with the propagation direction of the wave. In transversal mode, instead, atoms move perpendicularly to the propagation of the wave. The average phonon speed $\bar{v}$ is due to both vibration modes of the crystal lattice. For instance, in crystalline silicon, the polarization vectors computed in [28] are generally neither parallel nor perpendicular to the wave vector, and only few phonon modes are distinctively longitudinal or transversal.

Moving through the crystal lattice, phonons undergo two different types of scattering [29]: (i) normal scattering, conserving the phonon momentum; (ii) resistive scattering, in which the phonon momentum is not conserved. The frequencies $\nu_{N}$ and $\nu_{R}$ of normal and resistive phonon scatterings determine the characteristic relaxation times $\tau_{N}=\frac{1}{\nu_{N}}$ and $\tau_{R}=\frac{1}{\nu_{R}}$. It is worth noticing that the relaxation time of normal scatterings is related to the mean free path of phonons by the relation $\ell =\sqrt{\frac{9}{5}\frac{\kappa \tau_{N}}{c_{v}}}$. When ℓ≃L, as it happens at nanoscale, phonons can interact with other phonons or with the elements of the crystal lattice in any point of the conductor, i.e., they undergo nonlocal interactions. For that reason, the last term in Equation (2) is said to be representative of nonlocal effects.

When both relaxation times are not negligible, we face the heat equation (2). Since Equation (2) is parabolic, propagation of thermal disturbances is not possible. Heat waves take over, in fact, when $\nu_{R}$ remains finite while $\nu_{N}$ grows to infinity, letting $\tau_{N}$ tend to zero. For vanishing $\tau_{N}$, i.e., for vanishing *ℓ*, Equation (2) reduces to the Maxwell-Cattaneo hyperbolic equation [30]

(3) $$\tau_{R}\frac{\partial q}{\partial t}+q=-\lambda \nabla T,$$

which, in turn, yields the classical Fourier law in the absence of relaxation effects.

Since $\tau_{R}$, λ and *ℓ* are supposed to be constants, Equation (2) is linear. As a consequence, it does not take into account the non-linear effects, which instead are usual at the micro/nanoscale. In fact, at very small scales, even small differences in temperature may produce strong gradients, so that the second-order terms that contain the product of the gradient of the heat flux with another gradient or with the heat flux itself are no longer negligible. Extensions of Equation (2) to the non-linear regime can be obtained within the frame of Extended Irreversible Thermodynamics (EIT) [22,23,24,27,31] by using the first 4 equations of the system of phonon hydrodynamics and letting the heat flux and its first-order gradient to enter the state space [31,32].

A different approach, also inspired by kinetic theory, consists in introducing a scalar internal variable that plays the role of a semiempirical nonequilibrium temperature [23,33,34], whose gradient is proportional to the heat flux. Such a temperature can be defined within the frame of the Maxwell approach to kinetic theory, beyond the hypothesis of local equilibrium. In such a way, the Guyer-Krumhansl equation is obtained by postulating a partial differential equation for the semiempirical temperature.

The easiest extension of the Guyer-Krumhansl equation to nonlinear regimes takes the form [23]

(4) $$\tau_{R}\frac{\partial q}{\partial t}+q=-\lambda \nabla T+\mu q\cdot \nabla q+\ell^{2}\left(\nabla^{2}q+2\nabla \nabla \cdot q\right),$$

where $\mu =2\tau_{R}/\varrho c_{v}T$.

In the present paper, we use Equation (4), which includes the genuinely nonlinear term μq·∇q and the nonlocal term $\ell^{2}\left(\nabla^{2}q+2\nabla \nabla \cdot q\right)$, to describe the consequences of nonlinear and nonlocal effects on the heat flux rectification. To achieve that task, we compare the order of magnitude of the last two terms in the right-hand side of Equation (4), depending on the order of magnitude of the direct heat flux and of the radiative heat supply.

## 3. The Physical Model

Here we consider a one-dimensional rigid heat conductor in the steady state. Furthermore, Equation (4) becomes

(5) $$q=-\lambda T_{,x}+\mu qq_{,x}+3\ell^{2}q_{,xx},$$

wherein *x* is the spatial coordinate of the points of of the conductor, and $f_{,x}\equiv \partial f/\partial x$. Equation (5) is coupled with the local energy balance, which in the steady state reads

(6) $$q_{,x}=r,$$

with *r* as the external rate of energy provided to the system. For the sake of concision, since the system is rigid, we have included the mass density in *r* which, from now on, denotes the energy per unit volume and unit time. Here and in the following, we suppose r≠0. Such an hypothesis is fundamental in our investigation because of the following motivations:

- *r* allows the tuning of the heat-flux rectification;
- by Equations (5) and (6) it follows that the nonlinear and nonlocal terms in Equation (5) are present if, and only if, r≠0, otherwise, q is constant along the system, and the second and third terms of Equation (5) vanish.

From the physical point of view, r≠0 means that the nanowire under consideration is heated in two different ways, namely, by:

1. heating its interior points by a given heat supply per unit volume and time;
2. applying a direct or reverse heat flux on its boundary.

The thermal conductivity will be assumed to depend on composition and temperature, while for the material parameters μ and $\ell^{2}$ we will use their constant values at T=300K (room temperature).

To proceed further, we make the approximations $q_{,x}≃q/L$ and $q_{,xx}≃q/L^{2}$, with *L* as the length of the conductor. Thus, the coupling of Equations (5) and (6) yields

(7) $$q\left[1-\mu r-3(\ell^{2}/L^{2})\right]=-\lambda T_{,x}.$$

Equation (7) takes the form of the classical Fourier law if the effective thermal conductivity $\lambda_{eff}\equiv \lambda /\left[1-\mu r-3(\ell^{2}/L^{2})\right]$ is introduced. Such a conductivity makes physical sense if, and only if, the additional constraint $1-\mu r-3(\ell^{2}/L^{2})>0$ holds.

The material parameters $\mu =2\tau_{R}/\varrho c_{v}T$ and Kn=ℓ/L (Knudsen number) entering Equation (7) are chosen as follows.

We use the experimental data in Ref. [24], Tables 1.1 and 1.2 therein, for the volumetric heat capacity $C_{v}$, the thermal conductivity λ, and the phonon mean speed $\bar{v}$ for bulk Si and bulk Ge at room temperature (300K).

Furthermore, the phonon mean free paths can be calculated through the relation $\ell =3\lambda /C_{v}\bar{v}$. It yields $\ell_{\mathrm{Si}}=8.05\times 10^{-8}m$, and $\ell_{\mathrm{Ge}}=5.83\times 10^{-8}m$.

It seems important to wonder if the value of $\ell_{\mathrm{Si}}$ calculated through this procedure is in accordance with the results obtained in [35], wherein it is proved that phonons with mean free paths smaller than 1micron considerably contribute to heat transport. To answer that question, we note that the value of $\lambda_{\mathrm{Si}}$ used for calculating $\ell_{\mathrm{Si}}$ is 128.03W/mK. On the other hand, from Figure 6 in ref. [35], it can be seen that the phonons with mean free path in the interval 100–300 nm produce a differential thermal conductivity smaller than 25W/mK, while the phonons with mean free path in the interval 50–100 nm produce a differential thermal conductivity between 50W/mK and 105W/mK, which is close to that used for our calculation. Thus, we conclude that the value of $\ell_{\mathrm{Si}}$ obtained in the present paper is acceptable and in accordance with the results in [35].

Once the mean free paths have been calculated, the relaxation times of bulk silicon and germanium can be calculated as $\tau_{\mathrm{Si}}=\ell_{\mathrm{Si}}/{\bar{v}}_{\mathrm{Si}}=2.78\times 10^{-11}$ s, and $\tau_{\mathrm{Ge}}=\ell_{\mathrm{Ge}}/{\bar{v}}_{\mathrm{Ge}}=3.31\times 10^{-11}$ s. Finally, the Matthiessen rule allows to estimate the relaxation time as

$$\frac{1}{\tau_{R}}=\frac{c}{\tau_{\mathrm{Si}}}+\frac{1-c}{\tau_{\mathrm{Ge}}}.$$

We make our computation at c=1/2, so that $\tau =1.51\times 10^{-11}$ s.

For mass density, we use the data in ref. [36], which yield the following expression of ϱ as function of the composition

$$\varrho \left(c\right)=\left(2.329+1.746c-0.499c^{2}\right)\times 10^{3}\;\;\mathrm{Kg}/m^{3}.$$

For c=1/2 it yields $\varrho =3.95\times 10^{3}\mathrm{Kg}/m^{3}$. Analogously, for the specific heat $c_{v}$ we take the function [36],

$$c_{v}=\left(0.7+0.04c\right)\times 10^{3}\;\;J/\left(\mathrm{Kg}K\right),$$

getting so $c_{v}=0.72\times 10^{3}\;\;J/\left(\mathrm{Kg}K\right)$. Thus

$$\mu =2\tau /\varrho c_{v}T=0.35\times 10^{-19}m^{3}/W.$$

For the nonlocal term $3\ell^{2}/L^{2}$ we use the results in [16], wherein the Knudsen number Kn=ℓ/L has been estimated to be Kn=0.4. Then we get $3{\mathrm{Kn}}^{2}=0.48$. In this way, Equation (7) can be rewritten as

(8) $$q\left[1-(0.35\times 10^{-19})\;\;r-0.48\right]=-\lambda T_{,x}.$$

For given values of *q*, the value of *r* is determined by the relation $r=q_{,x}≃q/L$.

Thus, for $q=10^{12}W/m^{2}$, we get $r≃q/L=10^{19}W/m^{3}$ and μr≃0.35. In this way, the nonlinear term and the nonlocal term in Equation (8) have the same order of magnitude and both show their effects. The generalized Fourier law Equation (8) becomes $q=-\left(1/0.17\right)\lambda T_{,x}$.

For $q=10^{11}W/m^{2}$, instead, then μr≃0.035, and the generalized Fourier law becomes $q=-\left(1/0.485\right)\lambda T_{,x}$. In such a case, the nonlocal effects are stronger than the nonlinear ones.

Finally, for $q=10^{10}W/m^{2}$, then μr≃0.0035, and the generalized Fourier law becomes $q=-\left(1/0.5165\right)\lambda T_{,x}$. In such a case, the nonlinear effects are still present but small with respect to the nonlocal ones, which are predominant. The same is true for $q=10^{9}W/m^{2}$, which yields μr≃0.00035 and $q=-\left(1/0.51965\right)\lambda T_{,x}$.

Thus, we can say that for the system at hand, if the heat flux obeys Equation (5), the nonlocal effects are always present, while the nonlinear effects are present and have the same order of magnitude as the nonlocal ones if $q=10^{12}W/m^{2}$; are present but have a smaller order of magnitude if $q=10^{11}W/m^{2}$; are present but are small if $q=10^{10}W/m^{2}$ or $q=10^{9}W/m^{2}$. Finally, for $q\leq 10^{8}W/m^{2}$ the nonlinear effects can be considered negligible.

It is worth noting that the values $q=10^{11}W/m^{2}$, and $q=10^{12}W/m^{2}$, seem to be too strong for realizable experiences, so that our numerical experiments will be focused on values of the heat flux in the range $\left[10^{7}W/m^{2}-\;10^{10}W/m^{2}\right]$.

We note that the values $q\geq 10^{13}W/m^{2}$ are not admissible, because in such a case $\lambda_{eff}<0$. The existence of forbidden values of the heat flux is frequent in the experiments, and is explained by admitting the existence of the so-called flux limiters [37].

For L=3mm and $q=10^{12}W/m^{2}$, we have $r≃10^{15}W/m^{3}$ and $\mu r≃0.11\times 10^{-4}$. Moreover, $\mathrm{Kn}=40\times 10^{-9}m/3\times 10^{-3}m≃13.33\times 10^{-6}$. Thus, $3{\mathrm{Kn}}^{2}≃5.33\times 10^{-4}$, so that both the nonlinear and nonlocal terms in Equation (5) are negligible. The same conclusion is true if either $q=10^{11}W/m^{2}$, or $q=10^{10}W/m^{2}$. Finally, the forbidden values of the heat flux [37] are, in such a case, $q\geq 10^{17}W/m^{2}$.

As a conclusion, we can say that for the model at hand, for systems of length L=100nm nonlocal effects are always present, while nonlinear effects are not negligible only for some values of *q*. For systems of length L=3mm, instead, both nonlinear and nonlocal effects are always negligible. Hence, the occurrence of nonlocality and nonlinearity can be regarded as a size effect that manifests itself at the nanoscale and disappears at the macroscopic scale.

## 4. Results and Discussion

In the present section, we show the possible consequences of the nonlinearity and nonlocality of the heat equation on the rectification of the heat current, for physically acceptable values of *q*. For a nanowire of length L=100nm, in the light of the conclusions of Section 3, and in order to consider situations that are experimentally realizable, we restrict our investigation to values of the heat flux in the interval $\left[10^{7}W/m^{2}-\;10^{10}W/m^{2}\right]$.

For $q=10^{7}W/m^{2}$, and $q=10^{8}W/m^{2}$ the nonlinear effects are too small, and have been neglected. For $q=10^{9}W/m^{2}$, and $q=10^{10}W/m^{2}$, we have taken into account the nonlinear effects too.

**Figure 3:**
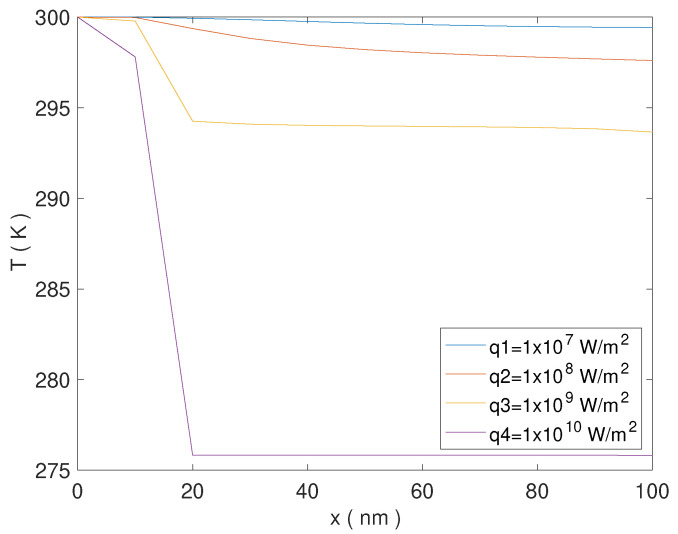
Temperature profiles for different values of the heat flux, at T=300K and for L=100nm.

The temperature profiles are shown in Figure 3, while the corresponding values of the rectification coefficient and of $q,r,\mu r,q_{eff},T_{H},T_{C}$, are shown in Table 1.

**Figure 4:**
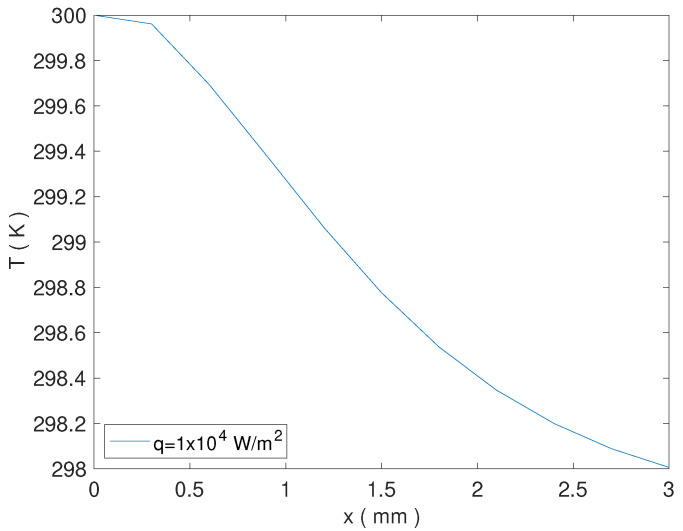
Temperature profile for $q=10^{4}$, at T=300K and for L=3mm.

For L=3mm we obtained that rectification of the heat flux is present for $q=10^{4}W/m_{2}$. The temperature profile is shown in Figure 4, while the corresponding value of the rectification coefficient and of $q,r,\mu r,q_{eff},T_{H},T_{C}$, are shown in Table 2.

We note that, in such a case, the qualitative behavior is similar to that obtained in Ref. [8] for thermoelastic solids with $c={\left(x/L\right)}^{2}$ (see Figure 5 therein).

For $q=10^{7}W/m^{2}$, and $q=10^{8}W/m^{2}$, as consequence of nonlocality the direct heat flux results multiplied by a reduction factor R≈0.52, which changes appreciably the heat conduction with respect to the case in which the nonlocal effects are not present (see Ref. [15] and Figure 2 therein for a comparison). The two temperature profiles in Figure 3 have a similar qualitative behavior but different values, determined by the different values of the applied heat flux (see blue and red lines in Figure 3). Finally, we note that both curves are regular, which is further evidence of the absence of nonlinear effects.

For $q=10^{9}W/m^{2}$ the reduction factor is R≈0.51965. However, the heat flux applied is 10 or 100 times stronger with respect to the previous cases, so that the temperature profile changes again (see yellow line in Figure 3).

Finally, for $q=10^{10}W/m^{2}$ the reduction factor is R≈0.5165, while the heat flux applied is 10 times stronger with respect to the last case. The combination of both these factors produces a drastic reduction in the values of the temperature (see the violet line in Figure 3).

The temperature profiles corresponding to the two last cases are nonregular (because of the nonlinearity of the heat equation) and present different behavior along the conductor. A very moderate (or moderate) decrease in the first part of the conductor close to the hottest end, then a strong reduction in a narrow strip of a few nanometers, and finally a constant value until to the coldest end. In both cases, the temperature drastically decreases at x≈18nm, which corresponds to c=0.18. At x≈20nm, which corresponds to c=0.20, the temperature starts to remain constant. We can regard such a distance as a kind of penetration depth of the direct heat flux, which beyond this distance is unable to produce any temperature gradient. On the other hand, in the rigid conductor considered in Ref. [15], of the same length with the same composition and the same dependency on *c* of the thermal conductivity, such a behavior is not present, and the temperature is continuously decreasing along the conductor (see Figure 2 therein). Thus, we suppose that such a phenomenon is due to the damping of the heat flux, which produces a temperature gradient only in a limited part of the conductor, close to the end of application.

## 5. Conclusions

In ref. [15] we have investigated heat rectification in a nanowire of length L=100nm subjected to a heat flux $q=10^{5}W/m^{2}$. We obtained a rectification coefficient R=2.77. A comparison with the results in Table 1 shows that the increment of the intensity of the heat flux, together with the presence of nonlinear and nonlocal effects, gives rise to a reduction of R. A comparison with the results in [38] confirms such a situation. Therein, the rectification coefficient for ${\mathrm{Si}}_{1-c}{\mathrm{Ge}}_{c}$ alloys has been calculated under the hypothesis of the validity of the Fourier law. For the composition used in the present paper for the calculation of the material constants, namely c=0.5, the authors find R=1.6, which is higher with respect to the values found in Section 4. In the same paper, for different *c*, values of R ranging up to 3.41 have been obtained. Thus, the drastic reduction of the rectification coefficient in the present model is evident.

Furthermore, a comparison of the first two lines of Table 1 with the last two lines of Table 1 shows that nonlinear effects produce a strong reduction of R. In particular, for $q=10^{9}W/m^{2}$ and $q=10^{10}W/m^{2}$, R is very close to zero, so that we conclude that heat can flow only from germanium to silicon but not from silicon to germanium. Such a type of system is called a thermal diode. Thus, the present results can provide useful information for thermal-diode design. It is worth mentioning that recently the possibility of using Si/Ge nanowires as thermal diodes has been investigated in [39,40].

On the other hand, the result in Table 2 shows that, although rectification can be enhanced at the nanoscale, it is present at the macroscale too. Since both nonlinear and nonlocal effects are negligible for such a system, rectification is due to the varying composition only.

In future investigations, we aim at analyzing nonlocal and nonlinear effects in the more usual situation of a system heated on its boundary only. To study non-local effects with heat given only at the edges, one has to go in two dimensions, by considering, for instance, one of the following cases:

1. radial heat flux in a cylindrical system;
2. Poiseuille flow in a plane system or along a cylindrical system.

Furthermore, in the light of the results shown in Section 4, it would be interesting to explore the possibility of tuning the penetration depth of the direct heat flux in order to determine a zone of the rigid conductor where the temperature is constant.

## Figures and Tables

**Table 1:** Rectification coefficient and corresponding values of $q,r,\mu r,q_{eff},T_{H},T_{C}$, at T=300K in a ${\mathrm{Si}}_{c}{\mathrm{Ge}}_{1-c}$ nanowire of length L=100nm.

$q\;(W/m^{2})$	$r\;(W/m^{3})$	μr	$q_{\mathrm{eff}}\;(W/m^{2})$	R	$T_{H}\;\;\;\left(K\right)$	$T_{C}\;\;\;\left(K\right)$
$1\times 10^{7}$	$10^{14}$	$0.35\times 10^{-5}$	$0.52\times 10^{7}$	0.782	300	299.42
$1\times 10^{8}$	$10^{15}$	$0.35\times 10^{-4}$	$0.52\times 10^{8}$	0.326	300	297.60
$1\times 10^{9}$	$10^{16}$	$0.35\times 10^{-3}$	$0.51965\times 10^{9}$	0.087	300	293.65
$1\times 10^{10}$	$10^{17}$	$0.35\times 10^{-2}$	$0.5165\times 10^{10}$	0.034	300	275.82

**Table 2:** Rectification coefficient and corresponding values of $q,r,\mu r,q_{eff},T_{H},T_{C}$, at T=300K in a ${\mathrm{Si}}_{c}{\mathrm{Ge}}_{1-c}$ wire of length L=3mm.

$q\;(W/m^{2})$	$r\;(W/m^{3})$	μr	$q_{\mathrm{eff}}\;(W/m^{2})$	R	$T_{H}\;\;\;\left(K\right)$	$T_{C}\;\;\;\left(K\right)$
$1\times 10^{4}$	$\frac{1}{3}\times 10^{7}$	$0.1155\times 10^{-12}$	$1\times 10^{4}$	0.702	300	298.00

## Data Availability

Not applicable.

## Appendix A. Dependency of Thermal Conductivity on Composition and Temperature

As we said above, in the analysis of Equation (7), we suppose that λ depends on composition and temperature, namely λ=λ(c,T). The present section is devoted to the determination of such a function. We will proceed in two steps. The first step consists in obtaining a best-fit curve of the function λ=λ(c) by the experimental values of thermal conductivity of silicon-germanium alloys as a function of the composition [19,20]. The disposition of such experimental points in the plane (c,λ(c)) is well represented by the following sum of two exponential functions

(A1) $$\lambda \left(c\right)=\Gamma_{1}\left(M,N,P,Q\right)e^{Mc^{2}+Nc}+\Gamma_{2}\left(M,N,P,Q\right)e^{Pc^{2}+Qc},$$

with *M*, *N*, *P*, *Q* independent parameters, and $\Gamma_{1}\left(M,N,P,Q\right)$ and $\Gamma_{2}\left(M,N,P,Q\right)$ regular functions of them.

In order to determine the best value of *M*, *N*, *P*, *Q* by a Non Linear Regression Method (NLRM) [41], we must distinguish between the direct case, in which the heat flux is directed from Si to Ge, and the reverse case, in which the heat flux is directed from Ge to Si. Such a distinction is necessary because when the heat flux is directed from Si to Ge the heat carriers move through a region with a decreasing stoichiometric variable *c*, while when the heat flux is directed from Ge to Si, the heat carriers move into a region with an increasing stoichiometric variable *c*. Such a situation can be represented by the same heat flux applied to two different materials, namely, ${\mathrm{Si}}_{c}{\mathrm{Ge}}_{1-c}$ and ${\mathrm{Ge}}_{c}{\mathrm{Si}}_{1-c}$. For the first material the functions $\Gamma_{1}\left(M,N,P,Q\right)$ and $\Gamma_{2}\left(M,N,P,Q\right)$ are determined by the constraint $\lambda \left(0\right)=\lambda_{\mathrm{Ge}}$ and $\lambda \left(1\right)=\lambda_{\mathrm{Si}}$, for the second material by the constraint $\lambda \left(0\right)=\lambda_{\mathrm{Si}}$ and $\lambda \left(1\right)=\lambda_{\mathrm{Ge}}$. In Table A1 are shown the values of thermal conductivity for pure Si and pure Ge at three reference temperatures. Such values are fundamental to our next computation.

**Table A1:** Thermal conductivity of pure Si and pure Ge, at T=300K, T=400K and T=500K for L=100nm.

Temperature(K)	$\lambda_{\mathrm{Si}}\;\;\;\left({\mathrm{Wm}}^{-1}K^{-1}\right)$	$\lambda_{\mathrm{Ge}}\;\;\;\left({\mathrm{Wm}}^{-1}K^{-1}\right)$
T=300	43.58	23.52
T=400	38.16	20.54
T=500	33.40	18.19

Thus, by the procedure described above, in the direct case we get

(A2) $$\lambda \left(c\right)=\frac{\lambda_{Si}-\lambda_{Ge}e^{P+Q}}{e^{M+N}-e^{P+Q}}e^{Mc^{2}+Nc}+\frac{-\lambda_{Si}+\lambda_{Ge}e^{M+N}}{e^{M+N}-e^{P+Q}}e^{Pc^{2}+Qc}.$$

wherein the material parameters *M*, *N*, *P*, and *Q* are given in Table A2 below.

**Table A2:** Values of the parameters in Equation (A2) for a ${\mathrm{Si}}_{c}{\mathrm{Ge}}_{1-c}$ alloy of length L=100nm.

Temperature(K)	*M*	*N*	*P*	*Q*
T=300	6.3805	−5.3627	252.5494	−251.9632
T=400	239.7700	−239.1857	6.3053	−5.2827
T=500	228.2427	−227.6759	6.2079	−5.1835

In the reverse case, instead, we get

(A3) $$\lambda \left(c\right)=\frac{\lambda_{Ge}-\lambda_{Si}e^{P+Q}}{e^{M+N}-e^{P+Q}}e^{Mc^{2}+Nc}+\frac{-\lambda_{Ge}+\lambda_{Si}e^{M+N}}{e^{M+N}-e^{P+Q}}e^{Pc^{2}+Qc},$$

with *M*, *N*, *P*, and *Q* given in Table A3 below.

**Table A3:** Values of the parameters in Equation (A3) for a ${\mathrm{Si}}_{1-c}{\mathrm{Ge}}_{c}$ alloy of length L=100nm.

Temperature(K)	*M*	*N*	*P*	*Q*
T=300	252.5491	−253.1354	6.3805	−7.3983
T=400	236.4300	−237.0146	6.3193	−7.3392
T=500	220.4237	−220.9902	6.2479	−7.2732

As far as the dependency on T is concerned, since we have no experimental data of λ as function of temperature for Si/Ge nanowires of length L=100nm, we look for a second-order approximation of it, in the neighborhood of the three reference temperatures. For $\Gamma_{1}\left(T\right)$ and $\Gamma_{2}\left(T\right)$, instead, we take the expressions used in Equations (A2) and (A3). Thus, for T=300K, up to the second order approximation, we get

$$M\left(T\right)=M\left(300\right)+a_{1}\left(T-300\right)+\frac{1}{2}a_{2}{\left(T-300\right)}^{2}.$$

Furthermore, Table A2 yields

M(300)=6.3805,

$$M\left(400\right)=6.3805+a_{1}\left(100\right)+a_{2}{\left(100\right)}^{2}=239.7700,$$

$$M\left(500\right)=6.3805+a_{1}\left(200\right)+a_{2}{\left(200\right)}^{2}=228.2427.$$

The linear system above allows us to calculate the coefficients $a_{1}$ and $a_{2}$. Moreover, the same procedure leads to the determination of the coefficients $b_{i}$, $d_{i}$ and $e_{i}$, i=1,2, entering the functions N(T), P(T), and Q(T), respectively. The values of the coefficients, both for the direct case (${\mathrm{Si}}_{c}{\mathrm{Ge}}_{1-c}$) and for the reverse case (${\mathrm{Ge}}_{c}{\mathrm{Si}}_{1-c}$), are shown in Table A4 and Table A5 below.

**Table A4:** Coefficients $a_{i}$, $b_{i}$, $d_{i}$ and $e_{i}$, for a ${\mathrm{Si}}_{c}{\mathrm{Ge}}_{1-c}$ alloy of length L=100nm.

$a_{1}$	$a_{2}$	$b_{1}$	$b_{2}$
3.5584	−0.0244	−3.5648	0.0245
$d_{1}$	$d_{2}$	$e_{1}$	$e_{2}$
−3.6931	0.0246	3.6997	−0.0246

**Table A5:** Coefficients $a_{i}$, $b_{i}$, $d_{i}$ and $e_{i}$ for a ${\mathrm{Si}}_{1-c}{\mathrm{Ge}}_{c}$ alloy of length L=100nm.

$a_{1}$	$a_{2}$	$b_{1}$	$b_{2}$
−0.1617	0.000011	0.1616	$-9.64\times 10^{-6}$
$d_{1}$	$d_{2}$	$e_{1}$	$e_{2}$
−0.0005	$-1.02\times 10^{-6}$	0.0005	$6.9\times 10^{-7}$

By this procedure, the function λ(c,T) can be calculated up to the second order of approximation in T.

A similar method can be applied for a ${\mathrm{Si}}_{c}{\mathrm{Ge}}_{1-c}$ alloy of length L=3mm. In Table A6, Table A7, Table A8, Table A9 and Table A10 below, we show the values of the parameters already shown in Table A1, Table A1, Table A2, Table A3, Table A4 and Table A5 for a nanowire of length L=100nm.

**Table A6:** Thermal conductivity (in ${\mathrm{Wm}}^{-1}K^{-1}$) of pure Si and pure Ge at T=300K, T=400K and T=500K for L=3mm.

Temperature(K)	$\lambda_{\mathrm{Si}}\;\;\;\left({\mathrm{Wm}}^{-1}K^{-1}\right)$	$\lambda_{\mathrm{Ge}}\;\;\;\left({\mathrm{Wm}}^{-1}K^{-1}\right)$
T=300K	149.95	77.95
T=400K	119.54	59.42
T=500K	92.01	48.08

**Table A7:** Values of the parameters in Equation (A2) for a ${\mathrm{Si}}_{c}{\mathrm{Ge}}_{1-c}$ alloy of length L=3mm.

Temperature(K)	*M*	*N*	*P*	*Q*
T=300K	4.8706	−3.76	109.4531	−108.9538
T=400K	91.8040	−91.3512	4.4159	−3.3127
T=500K	80.4957	−80.0740	4.0666	−2.9716

**Table A8:** Values of the parameters in Equation (A3) for a ${\mathrm{Si}}_{1-c}{\mathrm{Ge}}_{c}$ alloy of length L=3mm.

Temperature(K)	*M*	*N*	*P*	*Q*
T=300K	4.8707	−5.9814	109.4562	−109.9555
T=400K	4.4159	−5.5190	91.8040	−92.2568
T=500K	80.4998	−80.9215	4.0667	−5.1617

**Table A9:** Coefficients $a_{i}$, $b_{i}$, $d_{i}$ and $e_{i}$, for a ${\mathrm{Si}}_{c}{\mathrm{Ge}}_{1-c}$ alloy of length L=3mm.

$a_{1}$	$a_{2}$	$b_{1}$	$b_{2}$
1.3605	−0.0098	−1.3702	0.0098
$d_{1}$	$d_{2}$	$e_{1}$	$e_{2}$
−1.5738	0.0104	1.5829	−0.0105

**Table A10:** Coefficients $a_{i}$, $b_{i}$, $d_{i}$ and $e_{i}$ for a ${\mathrm{Si}}_{1-c}{\mathrm{Ge}}_{c}$ alloy of length L=3mm.

$a_{1}$	$a_{2}$	$b_{1}$	$b_{2}$
−0.3872	0.0076	0.3839	−0.0075
$d_{1}$	$d_{2}$	$e_{1}$	$e_{2}$
0.1738	−0.0070	−0.1699	0.0069
